# Genetic Determinants for Prediction of Outcome of Patients with Papillary Thyroid Carcinoma

**DOI:** 10.3390/cancers13092048

**Published:** 2021-04-23

**Authors:** Antónia Afonso Póvoa, Elisabete Teixeira, Maria Rosa Bella-Cueto, Rui Batista, Ana Pestana, Miguel Melo, Thalita Alves, Mafalda Pinto, Manuel Sobrinho-Simões, Jorge Maciel, Paula Soares

**Affiliations:** 1 Department of General Surgery, Centro Hospitalar de Vila Nova de Gaia/Espinho (CHVNG/E), 4434-502 Vila Nova de Gaia, Portugal; jpmaciel@ufp.edu.pt; 2 IPATIMUP—Instituto de Patologia e Imunologia Molecular da Universidade do Porto, 4200-135 Porto, Portugal; eteixeira@ipatimup.pt (E.T.); rbatista@ipatimup.pt (R.B.); apestana@ipatimup.pt (A.P.); jmiguelmelo@live.com.pt (M.M.); mafaldap@ipatimup.pt (M.P.); ssimoes@ipatimup.pt (M.S.-S.); 3 Cancer Signaling and Metabolism, i3S—Instituto de Investigação e Inovação em Saúde, 4200-135 Porto, Portugal; 4 Departament of Pathology, Faculdade de Medicina da Universidade do Porto, 4200-319, Porto, Portugal; 5 Department of Pathology, Parc Taulí Sabadell Hospital Universitari—Institut d’Investigació i Innovació Parc Taulí—I3PT—Universitat Autònoma de Barcelona, 08208 Barcelona, Spain; rbella@tauli.cat; 6 Department of Endocrinology, Centro Hospitalar Universitário de Coimbra,3000-075 Coimbra, Portugal; 7 Laboratório de Endocrinologia Molecular e Translacional—Departamento de Medicina, Universidade Federal de São Paulo, São Paulo 04039-032, Brazil; tha.alves@gmail.com; 8 Faculdade de Ciências da Saúde, Universidade Fernando Pessoa, 4200-253 Porto, Portugal

**Keywords:** thyroid cancer, papillary thyroid carcinoma, prognosis, BRAF, RAS, TERT, patient outcome, recurrent/persistent disease, structural disease, PTC-specific mortality

## Abstract

**Simple Summary:**

Aggressive metastatic disease is rare in papillary thyroid carcinoma (PTC), a neoplasia that usually carries an excellent prognosis. BRAF, RAS, and TERT promoter (TERTp) genes are altered in PTC, and their impact on patient outcomes remains controversial. We performed Sanger sequencing on a series of 241 PTCs to determine the role of genetic mutations (BRAF, RAS, and TERTp) in PTC patient outcomes. The implication of RAS^mut^ tumors remain uncertain in clinical terms. BRAF^mut^/TERTp^wt^ tumors were prone to be associated with local aggressiveness (recurrent, persistent/disease), whereas TERTp^mut^ tumors were predisposed to recurrent/persistent structural disease, and disease-specific mortality. Our results indicate that different molecular markers play a distinct role in predicting PTC patient outcomes.

**Abstract:**

Papillary thyroid carcinoma (PTC) usually presents an excellent prognosis, but some patients present with aggressive metastatic disease. BRAF, RAS, and TERT promoter (TERTp) genes are altered in PTC, and their impact on patient outcomes remains controversial. We aimed to determine the role of genetic alterations in PTC patient outcomes (recurrent/persistent disease, structural disease, and disease-specific mortality (DSM)). The series included 241 PTC patients submitted to surgery, between 2002–2015, in a single hospital. DNA was extracted from tissue samples of 287 lesions (primary tumors and metastases). Molecular alterations were detected by Sanger sequencing. Primary tumors presented 143 BRAF, 16 TERTp, and 13 RAS mutations. Isolated TERTp^mut^ showed increased risk of structural disease (HR = 7.0, *p* < 0.001) and DSM (HR = 10.1, *p* = 0.001). Combined genotypes, BRAF^wt^/TERTp^mut^ (HR = 6.8, *p* = 0.003), BRAF^mut^/TERTp^mut^ (HR = 3.2, *p* = 0.056) and BRAF^mut^/TERTp^wt^ (HR = 2.2, *p* = 0.023) showed increased risk of recurrent/persistent disease. Patients with tumors BRAF^wt^/TERTp^mut^ (HR = 24.2, *p* < 0.001) and BRAF^mut^/TERTp^mut^ (HR = 11.5, *p* = 0.002) showed increased risk of structural disease. DSM was significantly increased in patients with TERTp^mut^ regardless of BRAF status (BRAF^mut^/TERTp^mut^, log-rank *p* < 0.001; BRAF^wt^/TERTp^mut^, log-rank *p* < 0.001). Our results indicate that molecular markers may have a role in predicting PTC patients’ outcome. BRAF^mut^/TERTp^wt^ tumors were prone to associate with local aggressiveness (recurrent/persistent disease), whereas TERTp^mut^ tumors were predisposed to recurrent structural disease and DSM.

## 1. Introduction

Thyroid cancer (TC) is the most common endocrine malignancy worldwide, and its incidence has been remarkably increasing over the past three decades, particularly in developed countries [1,2,3,4]. According to GLOBOCAN 2018, TC accounts for 3.1% of all diagnosed cancer worldwide [5]. TC incidence was 18.6/100,000 in women and 4.1/100,000 in men in the same period [6], mainly due to one TC histotype, papillary thyroid carcinoma (PTC) [7,8]. The overall mortality rate for both genders was about 0.30/100,000 [6] (less than 5% at 10-year follow-up [9]), which remains low despite increasing TC incidence. While usually presenting an excellent prognosis, some PTC patients experience persistence/recurrence or metastatic disease [10,11], and patients with distant metastases (DM) display 45% disease-specific survival (DSS) at 10-year follow-up [12]. Presurgical prognostication is practically nonexistent [13]. Fine-needle aspiration biopsy (FNAB) is an important tool for TC diagnosis [13]. FNAB presents limitations, with 10–40% of cases being classified as indeterminate without providing any prognostic indication [14,15]. Several factors (such as gender and age, tumor invasion, namely angioinvasion and extrathyroidal extension, presence of metastases, response to treatment) have proven prognostic value in PTC [16]. Over the last years, initiation and progression of TC have been steadily associated with genetic/epigenetic events that lead to the activation of cellular signaling pathways, namely MAPK and PI3K-AKT [17,18,19]. Several studies have advanced the detection of alterations of genes or gene panels for PTC diagnosis and/or prognosis evaluation and patient management [20]. Mutations in BRAF, RAS, and TERT promoter (TERTp) genes have been described as altered in PTC by our group [10,21,22] and by others [20,23,24]. Yet, discordance exists on how these genes impact tumor behavior and patient outcome. A BRAF^V600E^ point mutation on exon 15 is frequently detected in PTC, accounting for more than 90% of all BRAF^mut^ in TC. BRAF^mut^ is present in about 45% PTCs [25] and has been associated, in some studies, with features of clinical aggressiveness such as older age, larger tumors, extrathyroidal extension (ETE), lymph node metastases (LNM), higher stage, and poorer prognosis [26,27]. The impact of BRAF^mut^ in PTC DM, lack of response to radioiodine (RAI) therapy, and mortality are still controversial [10,26,28,29,30]. The genetic significance of RAS^mut^ in TC remains an open question since point mutations are detected in all subtypes of thyroid nodules, from benign to anaplastic lesions [31,32]. Studies on RAS^mut^ and TC prognostication have shown controversial results so far. Some studies showed no association of RAS^mut^ with poorer prognosis [33], while others have reported an association with DM and poorer survival [34,35]. TERTp^mut^ was found in approximately 10% of PTC cases [4], being more frequent in aggressive PTC variants [22]. In TC, TERTp^mut^ has been associated with older age, larger tumors, ETE, higher tumor stage, DM, RAI therapy resistance, and patient mortality, by our group [10,21,22] and by others [36,37,38].

Beyond their utility for diagnostic purposes, further data are needed in order to use molecular markers as prognostic tools in TC. In this study, we decided to evaluate BRAF, RAS (NRAS, HRAS, and KRAS), and TERTp molecular status in a consecutive series of PTC patients in an attempt to assess the impact of gene status on patients’ outcome, namely recurrent/persistent disease, structural disease, and patient survival.

## 2. Materials and Methods

### 2.1. Patient Samples

The study was performed in a consecutive series of patients submitted to thyroid surgery at a single hospital (Centro Hospitalar de Vila Nova de Gaia e Espinho (CHVNG/E)), from January 2002 to December 2015 and diagnosed as PTC. Formalin-fixed, paraffin-embedded (FFPE) tissues were collected from institutional files. All tumor samples were reviewed by a single pathologist (MRB), according to the fourth edition of the World Health Organization classification of tumors of endocrine organs [39]. Inclusion criteria were PTC diagnosis in patients older than 18 years, followed for a minimum of two years (unless recurrence or disease-specific mortality (DSM) has occurred earlier), in which there were thyroid samples for histological re-evaluation. Following these criteria, 241 patients were included in the study. A total of 287 lesions were evaluated: besides 238 primary tumors, we reviewed 31 LNM at diagnosis, 16 locoregional recurrences (LR), and two DM, along with clinical information that was revised by a single physician (AAP).

The histological diagnosis of the 241 patients was the following: classical PTC (CPTC), follicular variant PTC (FVPTC), oncocytic variant PTC (OVPTC: Warthin-like PTC, classical oncocytic PTC, and follicular patterned oncocytic PTC) and aggressive variants of PTC (APTC: tall cell, hobnail, diffuse sclerosing and solid trabecular).

All procedures described in this study were in accordance with national and institutional ethical standards. The study was conducted in accordance with the Declaration of Helsinki, and the protocol was approved by the Ethics Committee of CHVNG/E (Project investigation 30/2016, 28 January 2016, Comissão de Ética do Centro Hospitalar de Vila Nova de Gaia/Espinho). According to Portuguese law, informed consent is not required for retrospective studies.

### 2.2. Patient Follow-Up and Risk Stratification

Patients were staged using the eighth edition of the American Joint Cancer Committee/Union for International Cancer Control (AJCC/UICC) staging system [40]. Risk stratification at the second year and at the end of follow-up was evaluated according to the 2015 American Thyroid Association (ATA) guidelines [11] for patients submitted to total thyroidectomy followed by RAI therapy. For patients that were not submitted to RAI or less than total thyroidectomy, risk stratification was performed using the system published by Momesso et al. [41].

According to 2015 ATA guidelines, no clinical evidence of disease (NED) at final follow-up was established if patients had thyroglobulin (Tg) levels that fit excellent response [11], no detectable Tg antibodies (TgAb), and no structural evidence of disease. Patients were classified as persistent disease whenever Tg values fit indeterminate or incomplete response (elevated basal or stimulated Tg values alone, without structural correlation), or there was any evidence of disease on cross-sectional imaging (ultrasonography (US), computed tomography (CT) scan), functional imaging (RAI scintigraphy or 2-[^18^F]fluoro-2-deoxy-d-glucose positron-emission tomography (2-^18^F-FDG-PET) scan), or biopsy-proven disease (cytology or histology). Several Tg assays were used with different functional sensitivities, reflecting the long study period. For the sake of simplification, the two patients who had positive TgAb, were included in the group of incomplete biochemical responses. Recurrence was defined if new biochemical, structural, and/or functional evidence of disease was detected following any period of NED.

Patients were considered to have a positive structural disease status if any of the following conditions were met: (1) positive cytology/histology, (2) highly suspicious lymph nodes or thyroid bed nodules in the neck US (hypervascularity, cystic areas, heterogeneous content, rounded shape, or enlargement on the follow-up), (3) findings in RAI scintigraphy, ^18^F-FDG-PET scans, or other cross-sectional imaging highly suspicious for metastatic disease. Recurrent/persistent disease (*n* = 57) was considered if patients had an indeterminate or incomplete response to treatment, whether it was only biochemical disease (*n* = 32) or biochemical and structural disease (*n* = 25). DSM was also an endpoint (patients dying of unrelated conditions had the final status determined based on data available before their demise).

### 2.3. DNA Extraction

DNA from FFPE tissues was retrieved from 10 µm sections after H&E guided careful microdissection. DNA extraction was performed using the GRS Genomic DNA Kit BroadRange (GRiSP Research Solutions, Porto, Portugal) following the manufacturer’s instructions. Quantitative and qualitative analysis of all samples was then performed by spectrophotometry using Nanodrop N-1000 Spectrophotometer for microvolume UV-Vis measurements (Thermo Scientific, Waltham, MA, USA).

### 2.4. PCR and Sanger Sequencing Analysis

Genetic characterization of the series of tumors regarding BRAF, RAS (NRAS, HRAS, and KRAS), and TERTp mutations was performed as previously reported [9,19,27]. Primer design was performed accounting for the most frequent regions mutated in PTC, namely, BRAF codon 600, NRAS codon 61, HRAS and KRAS codons 12, 13, and 61, and TERTp-124, and −146 regions.

Amplification of genomic DNA (25–50 ng) was achieved using the QIAGEN multiplex PCR kit (QIAGEN, Hilden, Germany) following the manufacturer’s instructions. The annealing temperature of 61 °C was established after protocol optimization for BRAF, NRAS, and TERTp segments amplification in the same reaction. HRAS and KRAS were screened separately by a touchdown PCR using the MyTaq HS Mix 2× Bioline PCR Kit (Meridian Bioscience, Cincinnati, OH, USA) following the manufacturer’s instructions. PCR amplification was confirmed in 1–2% agarose gel electrophoresis (GRS Agarose LE, GRiSP, Oporto, Portugal) and followed by PCR product purification. All tested hotspot mutations were sequenced by Sanger sequencing using the ABI Prism Big Dye Terminator kit v3.1 Cycle Sequencing (Fisher Scientific Applied Biosystems^®^, Portsmouth, NH, USA). After sequencing product precipitation, fragments were analyzed by capillary electrophoresis using the Applied Biosystems 3130/3130 ×l Genetic Analyzers (Foster city, CA, USA). For all genes, all detected mutations were validated by performing a new and independent analysis.

### 2.5. Statistical Analysis

Statistical analysis was performed with IBM SPSS Statistics version 25 (IBM, New York, NY, USA). Results were expressed in absolute frequency, percentage, mean ± standard deviation (Std), and median ± interquartile range (IR). Distribution analyses were performed using crosstabs analyses. Unpaired *t*-test and Mann–Whitney tests were applied whenever adequate. Disease-free survival (DFS) and DSM were accessed by Kaplan–Meier and log-rank tests. Age- and gender-adjusted models were created to evaluate the mutational status impact of evaluated genes in the outcomes: recurrent/persistent disease, structural disease, and DSM. Hazard ratios (HR) were assessed by Cox proportional hazard models. Statistical significance was accepted with a two-tailed *p*-value < 0.05.

## 3. Results

### 3.1. Description of the Patients

Out of 241 patients, 202 (83.8%) were female. The mean age at diagnosis was 52 ± 15.2 years (18–86 years), with 56.0% of the patients being <55 years. Median tumor size was 12 ± 10.0 mm (2–70 mm), with 97 (40.2%) primary tumors presenting a size ≤10 mm (microcarcinomas). The mean follow-up time was 7 ± 2.9 years (0.2–16.8 years). In three patients, the primary tumors were not available, and just their LNM was analyzed.

Characteristics of the patients and tumors are summarized in Table 1.

Two hundred twenty-five patients (93.4%) were at stage I, 12 patients (5.0%) were at stage II, and four patients (1.7%) were at stage IV.

During follow-up, 57 patients (23.7%) had recurrent/persistent disease, 25 patients (10.4%) had structural disease (LNM and/or DM) and nine patients (3.7%) had DSM.

### 3.2. Primary Tumors’ Genetic Characterization

The study of primary tumors demonstrated the presence of BRAF^mut^ in 143 (62.7%), TERTp^mut^ in 16 (7.3%), and RAS^mut^ in 13 (5.6%) patients.

All BRAF^mut^ cases presented with p.Val600Glu, which was present in all four histological subtypes, but more frequent in CPTC (Table 2). Three different TERTp^mut^ were found, −124 G > A mutation was detected in 12 cases (5.5%) from all four histological variants (Table 2), −146 G > A mutation was found in three PTC (1.4%), all CPTC, and one tandem −124/−125 G > A mutation was detected in an FVPTC. Of the 16 TERTp^mut^ tumors, 11 (5.1%) were concomitantly mutated for BRAF (BRAF^mut^/TERTp^mut^), and four (1.9%) were only TERTp^mut^ (BRAF^wt^/TERTp^mut^). One hundred and twenty (56.1%) tumors were BRAF^mut^ but not TERTp (BRAF^mut^/TERTp^wt^). A total of 13 (5.6%) RAS^mut^ were detected in primary tumors. p.Gln61Arg mutation was detected in all RAS genes (NRAS, HRAS, and KRAS) with a frequency of 3.5%, 2.3%, and 1.7%, respectively, mostly in follicular patterned tumors. One KRAS p.Gln61Arg mutation was concomitantly present with a −124 G > A TERTp^mut^.

The detailed univariate analysis of clinicopathological features in relation to combined gene mutations is presented in Supplementary Table S1.

### 3.3. Metastatic Lesions’ Genetic Characterization and Molecular Profile Concordance

The study of 31 LNM that were present at diagnosis demonstrated the presence of BRAF^mut^ in 17 LNM (54.8%) and TERTp^mut^ in three LNM (10.0%). Of those, two LNM (6.7%) were concomitantly BRAF^mut^/TERTp^mut^. The study of 16 cervical recurrences (14 recurrent LNM and two thyroid bed recurrences) revealed BRAF^mut^ in 12 lesions (75.0%), TERTp^mut^ in five lesions (33.3%), and being in three lesions concomitant BRAF^mut^/TERTp^mut^ (20.0%). The study of two DM demonstrated the presence of NRAS^wt^ in one lesion and one concomitant BRAF^mut^/TERTp^mut^ in the other lesion. Detailed information of metastatic lesions molecular profile is presented in Supplementary Table S2.

### 3.4. Molecular Alterations in Recurrent/Persistent Disease

**Figure 1:**
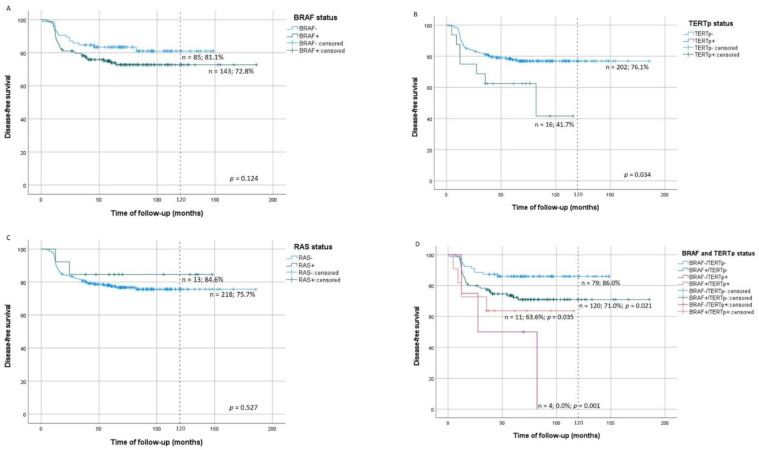
Kaplan–Meier curves of PTC of recurrent/persistent disease-free survival by tumor molecular status in comparison with wild type tumors; (**A**) BRAF status, (**B**) TERTp status, (**C**) RAS status, (**D**) combined BRAF and TERTp status in comparison to wild-type tumors; wt: wild-type; mut: mutated.

At 10-year evaluation, recurrent/persistent DFS was significantly lower in patients with TERTp^mut^ tumors (41.7%, *p* = 0.034). Patients with TERTp^mut^ tumors presented 2.3 times increased risk of recurrent/persistent disease (*p* = 0.04), but this association was lost when the model was adjusted for age and gender (*p* = 0.062) (Table 3). Recurrent/persistent DFS was not significantly different in patients with BRAF^mut^ or RAS^mut^ tumors from patients with BRAF^wt^ or RAS^wt^ tumors (Figure 1A–C; Table 2). Considering concomitant mutations, at 10-year evaluation, recurrent/persistent DFS was significantly lower in patients with BRAF^mut^/TERTp^wt^ (log-rank *p* = 0.021), BRAF^mut^/TERTp^mut^ (log-rank *p* = 0.035) and BRAF^wt^/TERTp^mut^ (log-rank *p* = 0.001) tumors than in patients with BRAF^wt^/TERTp^wt^ tumors (Figure 1D). The aforementioned concomitant mutations significantly increased the risk of recurrent/persistent disease in comparison to BRAF^wt^/TERTp^wt^ tumors (Table 3).

### 3.5. Molecular Alterations in Structural Disease

**Figure 2:**
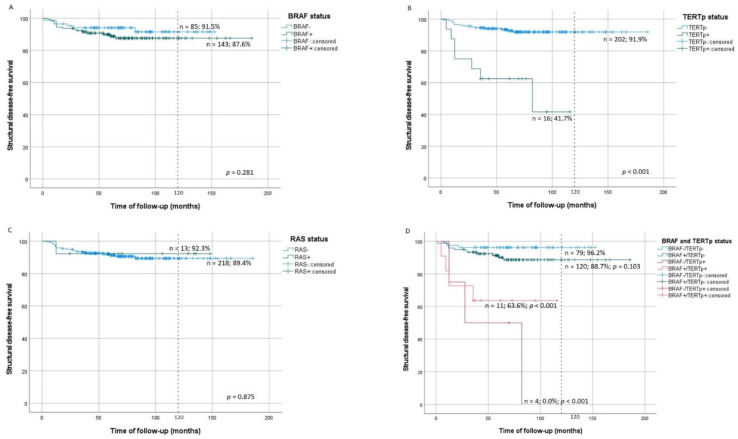
Kaplan–Meier survival curves of PTC structural disease-free survival (SDFS) by tumor molecular status in comparison with wild type tumors; (**A**) BRAF status, (**B**) TERTp status, (**C**) RAS status, (**D**) combined BRAF and TERTp status in comparison to wild-type tumors; wt: wild-type; mut: mutated.

Patients with TERTp^mut^ tumors had significantly lower structural disease-free survival (SDFS) (log-rank *p* < 0.001) than patients without TERTp^mut^ (Figure 2B) and presented significantly increased risk of structural disease, even when adjusted for age and gender (HR = 7.0, *p* < 0.001) (Table 4). SDFS was not significantly influenced by BRAF^mut^ or RAS^mut^ tumors (Figure 2A,C). Considering concomitant mutations, at 10-year evaluation, SDFS was significantly lower in patients with BRAF^mut^/TERTp^mut^ (log-rank *p* < 0.001) and BRAF^wt^/TERTp^mut^ (log-rank *p* < 0.001) tumors than in patients with BRAF^wt^/TERTp^wt^ tumors (Figure 2D). The aforementioned concomitant mutations significantly increased the risk of structural disease status both in unadjusted and adjusted models (Table 4). Patients with BRAF^mut^/TERTp^wt^ tumors did not display a significantly lower SDFS than BRAF^wt^/TERTp^wt^ tumors (log-rank *p* = 0.103) (Figure 2D).

### 3.6. Molecular Alterations in Disease-Specific Mortality

**Figure 3:**
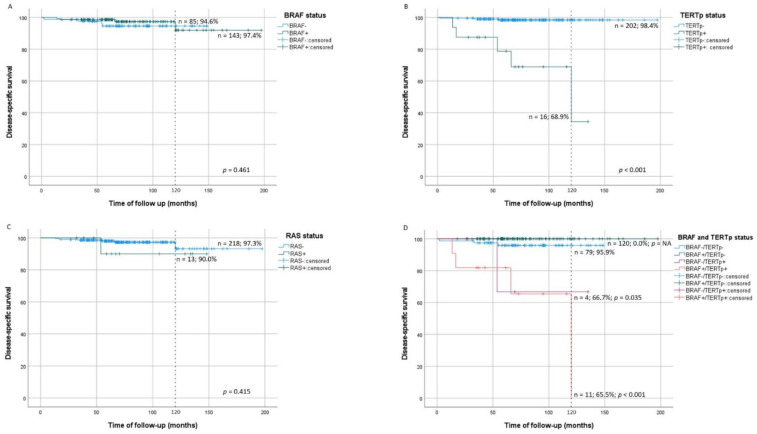
Kaplan–Meier survival curves of PTC disease-specific survival (DSS) by tumor molecular status in comparison with wild type tumors; (**A**) BRAF status, (**B**) TERTp status, (**C**) RAS status, (**D**) combined BRAF and TERTp status in comparison to wild-type tumors; wt: wild-type; mut: mutated.

DSM was significantly increased in patients with TERTp^mut^ tumors (log-rank *p* < 0.001) in comparison with patients without TERTp^mut^ (Figure 3B). When adjusted for gender and age at diagnosis, TERTp^mut^ significantly increased the risk of DSM (HR = 10.1, *p* = 0.010) (Table 5). DSM was not significantly increased in patients with BRAF^mut^ or RAS^mut^ tumors (Figure 3A,C). DSM was increased in patients with BRAF^mut^/TERTp^mut^ (log-rank *p* < 0.001) and BRAF^wt^/TERTp^mut^ (log-rank *p* = 0.035) tumors in comparison with patients with BRAF^wt^/TERTp^wt^ tumors (Figure 3D). There were no DSM events in patients with BRAF^mut^/TERTp^wt^ tumors (Table 5).

## 4. Discussion

In the present study, we aimed to investigate the role played by point mutations of BRAF, TERTp, and RAS (NRAS, HRAS, and KRAS) in the outcome of PTC patients (recurrent/persistent disease, structural disease status, and DSM). 

The most frequent alteration in our PTC series was, as expected, mutation of BRAF gene. BRAF^mut^ was also frequent in LNM at diagnosis and often detected in locoregional recurrences. All three RAS genes were found mutated in primary tumors, being overall the second most common alteration in PTC. The occurrence of TERTp^mut^, although less common than alterations of the other genes, was higher in recurrent and metastatic lesions, namely distant metastases, than in primary tumors, as hitherto shown by our group [10].

The role of BRAF^mut^ in the outcome of patients is still an open question. In our study, patients whose tumors had BRAF^mut^, regardless of the status of the other mutations, did not present a higher risk for any of the outcomes evaluated (recurrent/persistent disease, structural disease status, or DSM). In 2015, George et al. [42] reported a 92% frequency of BRAF^mut^ in a series of recurrent/persistent tumors. The increased incidence of BRAF^mut^ and the lack of a control group in this study did not allow to establish any association with recurrent/persistent disease [42]. In 2019, de Castro et al. [43] also did not find an association between BRAF^mut^ and recurrent/persistent disease, although their series contained 48% BRAF^mut^ tumors. This lack of association was corroborated by other studies [44,45,46]. Some studies have shown that older age and male gender are strong and independent risk factors for PTC—specific mortality in patients with BRAF^mut^ tumors, but not in patients with BRAF^wt^ tumors [27,47]. We did not find similar results in our series. The strength of BRAF^mut^ on patient outcomes may be overestimated in the aforementioned studies [27,47] since in them, no other molecular alterations, namely TERTp^mut^, were tested. As discussed below, our results indicate that the presence of TERTp^mut^ in PTC, and not of BRAF^mut^, indeed plays a major role in the outcome.

Several studies have demonstrated BRAF^mut^ association with PTC local invasiveness, recurrent/persistent disease, and LNM [30,48,49,50,51]; detection of BRAF^mut^ has led to advancing the idea that it may predict recurrence in low-risk CPTC patients [52]. Other studies suggested that BRAF^mut^ was associated with poorer outcomes, RAI treatment resistance, and DSM [26,29]. In contrast with this, it was suggested that isolated BRAF status has a limited role in guiding patient management [11], and other studies did not validate BRAF^mut^ impact on the outcome of patients [28,44,53,54]. In our analysis, isolated evaluation of BRAF^mut^, regardless of the status of the other mutations, converged with the limited role of this mutation in ascertain recurrent/persistent disease, structural disease, or DSM.

We detected mutations in all RAS genes. The most commonly described p.Gln61Arg mutation [55] was present in 13 primary tumors. It was shown that RAS^mut^ is present in all stages of thyroid neoplasia [55] ruling out the utilization of RAS^mut^ as a marker of malignancy. In a few studies, RAS^mut^ has been correlated with DM and poorer outcomes [34,56]. In our series, RAS^mut^ was not associated with a poorer prognosis; patients with RAS^mut^ had a stage I diagnosis, usually presenting excellent responses to therapy and were free of disease at the end of follow-up, except for one patient who had concomitant TERTp^mut^ and died from PTC. We can hypothesize that, in this patient, TERTp^mut^ was determinant in the outcome rather than the presence of KRAS^mut^. Of note, no RAS^mut^ was detected in locoregional metastases nor DM (Supplementary Table S2).

TERTp^mut^ has been consistently associated with poorer outcomes in PTC patients [57]. Some authors showed that recurrent/persistent disease was four times more frequent in patients with TERTp^mut^ than in patients with TERTp^wt^ tumors [58,59]. Whereas in another study, no significant increased risk of recurrent/persistent disease in patients with TERTp^mut^ tumors was reported [60]. The former finding fits with our own results; in the unadjusted analysis, we observed that patients with TERTp^mut^ tumors had twice the risk of recurrent/persistent disease; upon age and gender adjustment, there is still a suggested association (*p* = 0.062; Table 3).

In our study, TERTp^mut^ tumors were associated with significantly lower SDFS and significantly increased DSM. Furthermore, patients with TERTp^mut^ tumors presented a significantly increased risk of structural disease and of DSM, after adjustment for age and gender, suggesting that TERTp^mut^ may contribute to worse prognosis in PTC patients, as previously shown by our group [21]. Several studies have consistently associated TERTp^mut^ with DSM [21,22,42,46,57,58,59,61,62].

Given previous studies showing a frequent concomitant BRAF^mut^ and TERTp^mut^ in thyroid cancer [13,37,46,63,64,65], we tested the possible relationship of molecular status combinations and different outcomes. This analysis also aimed to evaluate a more trustworthy effect of each mutation separately.

Comparing with BRAF^wt^/TERTp^wt^ tumors, we observed that BRAF^mut^/TERTp^wt^ tumors were associated with an increased risk of recurrent/persistent disease. BRAF^wt^/TERTp^mut^ tumors were associated with an increased risk of recurrent/persistent disease, structural disease, and DSM. We are aware that the latter group (DSM) had few cases, and therefore these results should be interpreted with caution. Nevertheless, taking into account that TERTp^mut^ is far less common than BRAF^mut^ in PTC (less than 10% vs. 45%) [4,25] and that TERTp^mut^ is consistently associated with more aggressive forms of PTC and with increased risk of disease [22,53], we think that our results support the determinant role TERTp^mut^ in the outcome of PTC patients.

In our series, 11/16 TERTp^mut^ cases presented concomitant BRAF^mut^. We observed that BRAF^mut^/TERTp^mut^ significantly increased the risk of recurrent/persistent disease, structural disease, and DSM in comparison to BRAF^wt^/TERTp^wt^ tumors. In accordance with this, Kim et al. [61] showed that the presence of TERTp^mut^ was associated with increased mortality in PTC patients and further demonstrated that BRAF^mut^/TERTp^mut^ tumors were associated with worsened DSM in PTC patients in comparison to patients with isolated BRAF^mut^ tumors.

It was suggested that BRAF^mut^/TERTp^mut^ tumors presented a higher risk of worse outcomes in comparison with BRAF^wt^/TERTp^mut^ tumors [24,66]. In our study, we observed that DSM was similar in cases with BRAF^wt^/TERTp^mut^ tumors and BRAF^mut^/TERTp^mut^ tumors. Our results differ from those reported by Vuong et al. [67]; in the latter metanalysis, tumor aggressiveness varies according to isolated BRAF^mut^, isolated TERTp^mut^, or concomitant BRAF^mut^/TERTp^mut^. Vuong et al. [67] stratified PTC tumors’ aggressiveness in four groups with decreasing aggressiveness: BRAF^mut^/TERTp^mut^ > BRAF^wt^/TERTp^mut^ = BRAF^mut^/TERTp^wt^ > BRAF^wt^/TERTp^wt^. Xing et al. [24] proposed a synergistic role of concomitant BRAF^mut^/TERTp^mut^ on patients’ prognosis; in their series, BRAF^mut^/TERTp^mut^ tumors were associated with increased disease recurrence, higher than the sum of the two mutations alone, even when performing multivariable adjustments for the classical clinicopathologic risk factors. Xing et al. [24] hypothesized that a possible synergistic effect between the two mutations might occur, advancing that coexistence of the two mutations might be associated with increased expression of the TERT mRNA in PTC [24,64,65,66,68].

We observed that BRAF^wt^/TERTp^mut^ tumors had an increased risk of recurrent/persistent disease and structural disease in comparison with BRAF^mut^/TERTp^mut^ tumors. These results may indicate that TERTp^mut^, rather than BRAF^mut^, is the molecular alteration that confers higher aggressiveness to PTC. These results fit with our previous studies, in which we showed that PTC patients with TERTp^mut^ tumors had a significantly lower survival [10,21] at variance with the results obtained regarding BRAF^mut^. Similar findings were reported by Vuong et al. [53], who concluded that TERTp^mut^ was associated with unfavorable DSS and DFS, whereas BRAF^mut^ revealed an association with increased risk of recurrence but not with mortality, thus concluding that BRAF^mut^ usefulness to evaluate patient prognosis must be cautiously considered [53]. Gandolfi et al. [37] reported decreased survival in tumors TERTp^mut^ and BRAF^mut^/TERTp^mut^, but no association between BRAF^mut^ and DM or decreased survival was detected. BRAF^mut^ appears associated with local aggressiveness, while TERTp^mut^ was associated with distant metastasis that confers a dismal prognosis [10]. In accordance with our series, no significant differences in recurrent/persistent disease, structural disease, or DSM were observed when comparing patients with BRAF^mut^/TERTp^mut^ tumors and patients whose tumors were BRAF^wt^/TERTp^mut^ (Supplementary Figures S1–S3). The low number of tumors with BRAF^wt^/TERTp^mut^ genotype in our series indicates that a larger series is necessary to confirm our results.

## 5. Conclusions

We realize that our series has advantages and disadvantages; the positive aspect resides in the fact that it is a consecutive real-life series obtained from a single hospital and not a selected series. On the other hand, we are aware of the consequent limitations: few patients with DM and few patients with PTC-related death. As an example, having only nine patients dying from PTC disease makes the multivariate analysis for such output rather limited. These results should be evaluated with caution also due to the low number of patients with some genotypes.

Summing up, our results indicate that molecular markers can play a role in predicting the outcome of PTC patients. We have data supporting the importance of searching BRAF^mut^/TERTp^wt^ in terms of putative association with local aggressiveness (recurrent/persistent disease). Furthermore, we have observed a much more important result regarding the prognosis of patients with tumors presenting TERTp point mutations, given their increased risk to develop structural disease and DSM.

## Figures and Tables

**Table 1:** Series description: patient characteristics, tumor histology, staging, lymph node metastases, and patient outcome.

Patient Characteristics		*n*	(%)	*n* (Total)	Staging		*n*	(%)	*n* (Total)
**Age**				241	**T stage**				241
	<55 years	135	(56.0%)			T1a	96	(39.8%)	
	≥55 years	106	(44.0%)			T1b	97	(40.2%)	
**Gender**				241		T2	31	(12.9%)	
	Female	202	(83.8%)			T3a	7	(2.9%)	
	Male	39	(16.2%)			T3b	9	(3.7%)	
**Histology**		*n*	(%)	n (total)		T4a	1	(0.4%)	
**Nodule size**	Median ± IR	12.0 ± 10.00		238 ^a^	**N stage**				241
**Histological subtype**				238 ^a^		N0	207	(85.9%)	
	CPTC	156	(65.5%)			N1	34	(14.1%)	
	FVPTC	55	(23.1%)		**M stage**				241
	OVPTC	18	(7.6%)			M0	237	(98.3%)	
	AVPTC	9	(3.8%)			M1	4	(1.7%)	
**Necrosis**				237	**Stage**				241
	Absent	232	(97.9%)			SI	225	(93.4%)	
	Present	5	(2.1%)			SII	12	(5.0%)	
**Psammomas**				237		SIV	4	(1.7%)	
	Absent	155	(65.4%)		**Lymph node invasion and extranodal extension**		*n*	(%)	*n* (total)
	Present	82	(34.6%)		**Lateral compartment**				241
**Focality**				241	**Lymph node invasion**	Absent	225	(93.4%)	
	Unifocal	151	(62.7%)			Present	16	(6.6%)	
	Multifocal	90	(37.3%)		**Lateral compartment**				241
**Extrathyroidal extension**				240	**Extranodal extension**	Absent	236	(97.9%)	
	Absent/minimal	230	(95.8%)			Present	5	(2.1%)	
	Major	10	(4.2%)		**Patient outcome**		*n*	(%)	*n* (total)
**Lymphatic invasion**				237	**Recurrent/persistent**				241
	Absent	190	(80.2%)		**disease**	Absent	184	(76.3%)	
	Present	47	(19.8%)			Present	57	(23.7%)	
**Venous invasion**				239	**Structural disease**				241
	Absent	223	(93.3%)			Absent	216	(89.6%)	
	Present	16	(6.7%)			Present	25	(10.4%)	
**Resection margins**				240	**PTC related death**				241
	R0	209	(87.1%)			Alive or deceased by other causes	232	(96.3%)	
	R1	31	(12.9%)			Deceased of PTC	9	(3.7%)	

CPTC: classical PTC; FVPTC: follicular variant PTC; OVPTC: oncocytic variant PTC (includes Warthin-like, classical oncocytic PTC, and follicular patterned oncocytic PTC); AVPTC: aggressive variants of PTC (includes tall cell, hobnail, diffuse sclerosing, and solid/trabecular variants). ^a^ Three primary tumors were not available, only lymph node metastases.

**Table 2:** BRAF, TERTp, and RAS molecular status in primary tumors and distribution in the different PTC variants.

Gene(s)	Status	All *n* (%)	Histological Variants of Primary Tumors			
			CPTC	FVPTC	OVPTC	AVPTC
		*n* = 238 ^a^	*n* = 156	*n* = 55	*n* = 18	*n* = 9
**BRAF**	wt	85	39	33	9	4
	*p*.**Val600Glu**	**143 (62.7%)**	**110 (73.8%)**	**20 (37.7%)**	**8 (47.1%)**	**5 (55.6%)**
**TERTp**	wt	202	133	47	14	8
	**−124 G > A**	**12 (5.5%)**	**8 (5.6%)**	**2 (4.0%)**	**1 (6.7%)**	**1 (11.1%)**
	**−146 G > A**	**3 (1.4%)**	**3 (2.1%)**	0 (0.0%)	0 (0.0%)	0 (0.0%)
	**−124/-125 G > A**	**1 (0.5%)**	0 (0.0%)	1 (2.0%)	0 (0.0%)	0 (0.0%)
**BRAF/TERTp**	BRAF^wt^/TERTp^wt^	79	36	31	8	4
	**BRAF** ^**mut**^ **/TERTp** ^**wt**^	**120 (56.1%)**	**94 (67.1%)**	**16 (32.0%)**	**6 (40.0%)**	**4 (44.4%)**
	**BRAF** ^**wt**^ **/TERTp** ^**mut**^	**4 (1.9%)**	**2 (1.4%)**	**1 (2.0%)**	**1 (6.7%)**	0 (0.0%)
	**BRAF** ^**mut**^ **/TERTp** ^**mut**^	**11 (5.1%)**	**8 (5.7%)**	**2 (4.0%)**	0 (0.0%)	**1 (11.1%)**
**NRAS**	wt	223	152	47	15	9
	**p.Gln61Arg**	**8 (3.5%)**	**1 (0.7%)**	**5 (9.6%)**	**2 (11.8%)**	0 (0.0%)
**HRAS**	wt	127	75	36	10	6
	**p.Gln61Arg**	**3 (2.3%)**	0 (0.0%)	**3 (7.7%)**	0 (0.0%)	0 (0.0%)
**KRAS**	wt	117	67	35	10	5
	**p.Gln61Arg**	**2 (1.7%)**	**1 (1.5%)**	**1 (2.8%)**	0 (0.0%)	0 (0.0%)
**RAS** ^**b**^	wt	218	151	43	15	9
	**RAS** ^**mut**^	**13 (5.6%)**	**2 (1.3%)**	**9 (17.3%)**	**2 (11.8%)**	0 (0.0%)

CPTC: classical PTC; FVPTC: follicular variant PTC; OVPTC: oncocytic variant PTC (includes Warthin-like, classical oncocytic PTC, and follicular patterned oncocytic PTC); AVPTC: aggressive variants of PTC (includes tall cell, hobnail, diffuse sclerosing, and solid/trabecular variants). ^a^ Three primary tumors were not available, only lymph node metastases. ^b^ Represents RAS status if at least one of the RAS genes was sequenced; wt: wild-type; mut: mutated.

**Table 3:** Analysis of molecular status in the risk of recurrent/persistent disease.

Molecular Status	*n*	Recurrent/Persistent Disease Absent	Recurrent/Persistent Disease Present	Unadjusted HR (95% CI)	*p*-Value	Adjusted HR (95% CI)	*p*-Value *
**BRAF status**	228						
BRAF^wt^	85	70 (39.8%)	15 (28.8%)	1 (Reference)		1 (Reference)	
BRAF^mut^	143	106 (60.2%)	37 (71.2%)	1.6 (0.88–2.91)	0.128	−	−
**TERTp status**	218						
TERTp^wt^	202	157 (94.6%)	45 (86.5%)	1		1	
TERTp^mut^	16	9 (5.4%)	7 (13.5%)	2.3 (1.04–5.12)	**0.040**	2.2 (0.96–5.01)	0.062
**RAS status**	231						
RAS^wt^	218	168 (93.9%)	50 (96.2%)	1		1	
RAS^mut^	13	11 (6.1%)	2 (3.8%)	0.6 (0.16–2.61)	0.530	−	−
**BRAF and TERTp status **	214						
BRAF^wt^/TERTp^wt^	79	68 (41.7%)	11 (21.6%)	1		1	
BRAF^mut^/TERTp^wt^	120	87 (53.4%)	33 (64.7%)	2.2 (1.11–4.34)	**0.024**	2.2 (1.11–4.36)	**0.023**
BRAF^wt^/TERTp^mut^	4	1 (0.6%)	3 (5.9%)	6.5 (1.82–23.42)	**0.004**	6.8 (1.89–24.55)	**0.003**
BRAF^mut^/TERTp^mut^	11	7 (4.3%)	4 (7.8%)	3.4 (1.10–10.82)	**0.043**	3.2 (0.97–10.33)	0.056

*n*: number of cases; HR: Hazard ratio calculated by Cox Regression analysis; CI: Confidence interval; * Age- and gender-adjusted; *p*-value; wt: wild-type; mut: mutated.

**Table 4:** Analysis of molecular status in the risk of structural disease.

Molecular Status	*n*	Structural Disease Absent	Structural Disease Present	Unadjusted HR (95% CI)	*p*-Value	Adjusted HR (95% CI)	*p*-Value *
**BRAF status**	228						
BRAF^wt^	85	79 (38.3%)	6 (27.3%)	1 (Reference)		1 (Reference)	
BRAF^mut^	143	127 (61.7%)	16 (72.7%)	1.7 (0.65–4.26)	0.286	−	−
**TERTp status**	218						
TERTp^wt^	202	187 (95.4%)	15 (68.2%)	1		1	
TERTp^mut^	16	9 (4.6%)	7 (31.8%)	7.4 (3.01–18.23)	**<0.001**	7.0 (2.67–18.54)	**<0.001**
**RAS status**	231						
RAS^wt^	218	198 (94.3%)	20 (95.2%)	1		1	
RAS^mut^	13	12 (5.7%)	1 (4.8%)	0.9 (0.11–6.35)	0.875	−	−
**BRAF and TERTp status**	214						
BRAF^wt^/TERTp^wt^	79	76 (39.6%)	3 (13.6%)	1		1	
BRAF^mut^/TERTp^wt^	120	108 (56.3%)	12 (54.5%)	2.8 (0.79–9.90)	0.112	−	−
BRAF^wt^/TERTp^mut^	4	1 (0.5%)	3 (13.6%)	24.3 (4.89–120.80)	**<0.001**	24.2 (4.80–122.05)	**<0.001**
BRAF^mut^/TERTp^mut^	11	7 (3.6%)	4 (18.2%)	13.2 (2.93–59.05)	**0.001**	11.5 (2.40–55.60)	**0.002**

*n*: number of cases; HR: Hazard ratio calculated by Cox Regression analysis; CI: Confidence interval; * Age- and gender-adjusted; *p*-value; wt: wild-type; mut: mutated.

**Table 5:** Analysis of molecular status in the risk of disease-specific mortality.

Molecular Status	*n*	Disease-Specific Mortality Absent	Disease-Specific Mortality Present	Unadjusted HR (95% CI)	*p*-Value	Adjusted HR (95% CI)	*p*-Value *
**BRAF status**	228						
BRAF^wt^	85	81 (36.8%)	4 (50.0%)	1 (Reference)		1 (Reference)	
BRAF^mut^	143	139 (63.2%)	4 (50.0%)	0.6 (0.15–2.39)	0.466	-	-
**TERTp status**	218						
TERTp^wt^	202	199 (94.8%)	3 (37.5%)	1		1	
TERTp^mut^	16	11 (5.2%)	5 (62.5%)	23.9 (5.70–100.23)	**<0.001**	10.1 (1.76–58.41)	**0.010**
**RAS status**	231						
RAS^wt^	218	212 (94.6%)	6 (85.7%)	1		1	
RAS^mut^	13	12 (5.4%)	1 (14.3%)	2.4 (0.28–20.36)	0.429	-	-
**BRAF and TERTp status **	214						
BRAF^wt^/TERTp^wt^	79	76 (36.9%)	3 (37.5%)	1		1	
BRAF^mut^/TERTp^wt^	120	120 (58.3%)	0 (0.0%)	n.a.	n.a.	-	-
BRAF^wt^/TERTp^mut^	4	3 (1.5%)	1 (25.0%)	6.5 (0.67–63.31)	0.109	-	-
BRAF^mut^/TERTp^mut^	11	7 (3.4%)	4 (50.0%)	11.6 (2.59–52.09)	**0.001**	n.s.	n.s.

*n*: number of cases; HR (Hazard ratio) calculated by Cox Regression analysis; CI: Confidence interval; * Age- and gender-adjusted; *p*-value; wt: wild-type; mut: mutated.

## Data Availability

The data presented in this study are available on request from the corresponding author.

## Supplementary Materials

The following are available online at https://www.mdpi.com/article/10.3390/cancers13092048/s1, Figure S1: Kaplan–Meier curves of recurrent/persistent disease-free survival by concomitant BRAF and TERT promoter mutations in comparison to tumors mutated only for BRAF (E) and only for TERTp (F). Figure S2: Kaplan–Meier curves of structural disease-free survival by concomitant BRAF and TERT promoter mutations in comparison to tumors mutated only for BRAF (E) and only for TERTp (F). Figure S3: Kaplan–Meier curves of disease-specific survival by concomitant BRAF and TERT promoter mutations in comparison to tumors mutated only for BRAF (E) and only for TERTp (F). Table S1: Univariate analysis of clinicopathological features and combined gene mutations. Table S2: BRAF, TERTp, and RAS molecular status in lymph node metastases at diagnosis, locoregional recurrences, and distant metastases and distribution in the different PTC variants.
