# Supplementary material for: Genetic Determinants for Prediction of Outcome of Patients with Papillary Thyroid Carcinoma
Source: Cancers (Basel). 2021 Apr 23;13(9):2048. doi: 10.3390/cancers13092048 (PMC8122921; doi:10.3390/cancers13092048)
Supplement: Supplementary file 1 [file cancers-13-02048-s001.zip › cancers-1161982-Supplementary Table S1.pdf]

Supplementary Table S1: Univariate analysis of clinicopathological features and combined gene mutations

Clinicopathological features		A BRAFwt/TERTpwt n = 79 n (%)	B BRAFmut/TERTpwt n = 120 n (%)	C BRAFwt/TERTpmut n= 4 n (%)	D BRAFmut/TERTpmut n= 11 n (%)	p-values A… vs .B..	A… vs ..C.	A… vs …D	.B.. vs …D
**Patient characteristics**									
Age	Mean ± Std	51.1 ± 14.3	50.2 ± 15.2	55.8 ± 16.3	67.7 ± 14.6	0.691	0.610	**0.004**	**0.003**
Gender	Female	68 (86.1%)	102 (85%)	3 (75%)	7 (63.6%)	0.833	0.539	0.082	0.089
	Male	11 (13.9%)	18 (15%)	1 (25%)	4 (36.4%)				
**Histological characteristics**									
Nodule size	Median ± IR	12.0 ± 9.0	11.0 ± 9.0	26.5 ± 17.0	20.0 ± 16.0	0.158	**0.041**	0.267	0.057
Solid component	Absent	66 (83.5%)	117 (97.5%)	3 (75%)	9 (81.8%)	**<0.001**	0.530	1.000	0.056
	Present	13 (16.5%)	3 (2.5%)	1 (25%)	2 (18.2%)				
Inflammatory infiltrate	Absent	18 (22.8%)	6 (5%)	1 (25%)	0 (0%)	**<0.001**	1.000	0.112	1.000
	Present	61 (77.2%)	114 (95%)	3 (75%)	11 (100%)				
Fibrosis	Absent	11 (13.9%)	5 (4.2%)	0 (0%)	0 (0%)	**0.013**	1.000	0.347	1.000
	Present	68 (86.1%)	115 (95.8%)	4 (100%)	11 (100%)				
Venous invasion	Absent	70 (88.6%)	116 (96.7%)	3 (75%)	9 (81.8%)	**0.024**	0.408	0.619	0.080
	Present	9 (11.4%)	4 (3.3%)	1 (25%)	2 (18.2%)				
Lymph vessel invasion	Absent	69 (87.3%)	94 (78.3%)	2 (50%)	6 (54.5%)	0.106	0.098	**0.017**	0.130
	Present	10 (12.7%)	26 (21.7%)	2 (50%)	5 (45.5%)				
Nodule growth pattern	Expansive	59 (74.7%)	54 (45%)	4 (100%)	2 (18.2%)	**<0.001**	0.568	**<0.001**	0.115
	Infiltrative	20 (25.3%)	66 (55%)	0 (0%)	9 (81.8%)				
Minimal extrathyroidal extension	Absent	61 (78.2%)	52 (45.6%)	2 (50%)	3 (37.5%)	**<0.001**	0.228	**0.024**	0.729
	Minimal	17 (21.8%)	62 (54.4%)	2 (50%)	5 (62.5%)				
Gross extrathyroidal extension	Absent/ minimal	78 (98.7%)	114 (95%)	4 (100%)	8 (72.7%)	0.162	1.000	**0.005**	**0.028**
	Gross	1 (1.3%)	6 (5%)	0 (0%)	3 (27.3%)				
Resection margins	R0	72 (91.1%)	104 (86.7%)	3 (75%)	8 (72.7%)	0.334	0.339	0.102	0.200
	R1/R2	7 (8.9%)	16 (13.3%)	1 (25%)	3 (27.3%)				
Central lymph node metastasis	Absent	78 (98.7%)	101 (84.2%)	4 (100%)	8 (72.7%)	**0.001**	1.000	**0.005**	0.394
	Present	1 (1.3%)	19 (15.8%)	0 (0%)	3 (27.3%)				
Extranodal extension of central lymph node metastasis	Absent	79 (100%)	116 (96.7%)	4 (100%)	8 (72.7%)	0.153	-	**0.001**	0.013
	Present	0 (0%)	4 (3.3%)	0 (0%)	3 (27.3%)				
Lateral lymph node metastasis	Absent	74 (93.7%)	112 (93.3%)	4 (100%)	8 (72.7%)	0.153	1.000	0.055	0.050
	Present	5 (6.3%)	8 (6.7%)	0 (0%)	3 (27.3%)				
Extranodal extension of lateral lymph node metastasis	Absent	77 (97.5%)	120 (100%)	4 (100%)	8 (72.7%)	0.156	1.000	**0.012**	**<0.001**
	Present	2 (2.5%)	0 (0%)	0 (0%)	3 (27.3%)				
**Staging**									
T stage	T1-T2	73 (92.4%)	112 (93.3%)	4 (100%)	8 (72.7%)	0.802	1.000	0.077	0.050
	T3-T4	6 (7.6%)	8 (6.7%)	0 (0%)	3 (27.3%)				
N stage	N0	73 (92.4%)	100 (83.3%)	4 (100%)	8 (72.7%)	0.063	1.000	0.077	0.407
	N1	6 (7.6%)	20 (16.7%)	0 (0%)	3 (27.3%)				
M stage	M0	78 (98.7%)	120 (100%)	3 (75%)	9 (81.8%)	0.397	0.095	**0.038**	**0.006**
	M1	1 (1.3%)	0 (0%)	1 (25%)	2 (18.2%)				
Stage	SI	76 (96.2%)	114 (95%)	3 (75%)	6 (54.5%)	0.355	0.182	**<0.001**	**<0.001**
	SII	2 (2.5%)	6 (5%)	0 (0%)	3 (27.3%)				
	SIVB	1 (1.3%)	0 (0%)	1 (25%)	2 (18.2%)				
**RAI treatment**									
RAI mumber of treatments	1	53 (93%)	79 (85.9%)	2 (50%)	6 (66.7%)	0.184	**0.045**	**0.048**	0.151
	>1	4 (7%)	13 (14.1%)	2 (50%)	3 (33.3%)				
**Risk stratification**									
Risk stratification (RAI treated)	Low	32 (56.1%)	31 (33.7%)	0 (0%)	1 (11.1%)	**0.024**	**0.036**	**0.002**	**0.007**
	Intermediate	22 (38.6%)	55 (59.8%)	3 (75%)	4 (44.4%)				
	High	3 (5.3%)	6 (6.5%)	1 (25%)	4 (44.4%)				
Risk stratification (non-RAI treated)	Low	19 (86.4%)	18 (64.3%)	0 (0%)	1 (50%)	0.108	-	0.094	0.067
	Intermediate	3 (13.6%)	10 (35.7%)	0 (0%)	0 (0%)				
	High	0 (0%)	0 (0%)	0 (0%)	1 (50%)				
Global dynamic risk stratification (RAI treated)	Excelent	52 (91.2%)	90 (97.8%)	2 (50%)	5 (55.6%)	0.107	0.061	0.016	**<0.001**
	Incomplete	5 (8.8%)	2 (2.2%)	2 (50%)	4 (44.4%)				
Global dynamic risk stratification (non-RAI treated)	Excelent	21 (95.5%)	27 (96.4%)	0 (0%)	2 (100%)	1.000	-	1.000	1.000
	Incomplete	1 (4.5%)	1 (3.6%)	0 (0%)	0 (0%)				
