# Supplementary material for: Genetic Determinants for Prediction of Outcome of Patients with Papillary Thyroid Carcinoma
Source: Cancers (Basel). 2021 Apr 23;13(9):2048. doi: 10.3390/cancers13092048 (PMC8122921; doi:10.3390/cancers13092048)
Supplement: Supplementary file 1 [file cancers-13-02048-s001.zip › Povoa AA - Supplementary Figure S1.pdf]

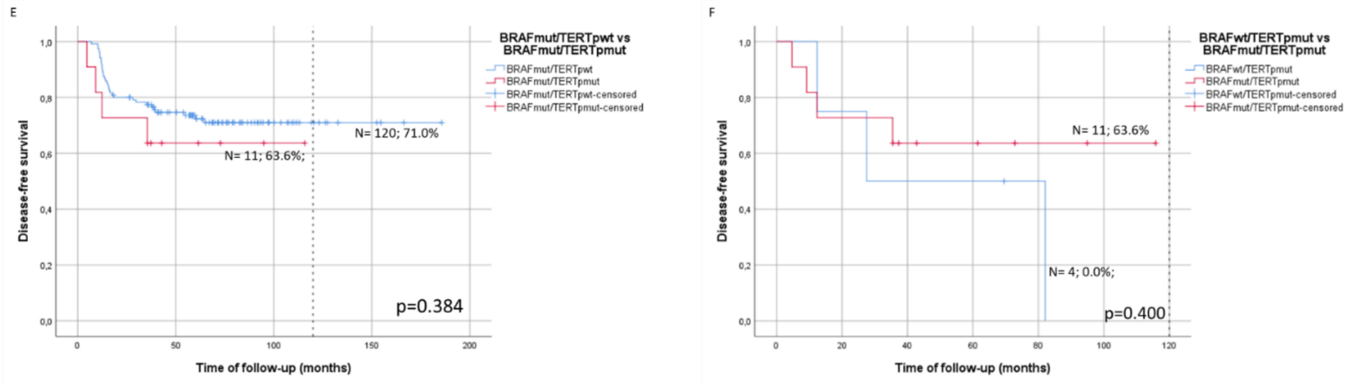

Supplementary Figure S1 – Kaplan-Meier curves of recurrent/persistent disease-free survival by concomitant BRAF and TERT promoter mutations in comparison to tumors mutated only for BRAF (E) and only for TERTp (F). wt: wild-type; mut: mutated.
