# Supplementary material for: Genetic Determinants for Prediction of Outcome of Patients with Papillary Thyroid Carcinoma
Source: Cancers (Basel). 2021 Apr 23;13(9):2048. doi: 10.3390/cancers13092048 (PMC8122921; doi:10.3390/cancers13092048)
Supplement: Supplementary file 1 [file cancers-13-02048-s001.zip › Povoa AA -Supplementary Table S2.pdf]

Supplementary Table S2 – BRAF, TERTp and RAS molecular status in lymph node metastases at diagnosis, locoregional recurrences and distant metastases and distribution in the different PTC variants.

**Gene(s)**	**Status**	**Lymph node metastases**	**Locoregional recurrences**	**Distant metastases**
		N=31	N=16	N=2[#]
**BRAF**	wt	14	4	0
	**p.Val600Glu**	**17 (54.8%)**	**12 (75.0%)**	**1 (n.a.)**
**TERTp**	wt	27	10	0
	**-124 G>A**	**2 (6.7%)**	**3 (20.0%)**	**1 (n.a.)**
	**-146 G>A**	**1 (3.3%)**	**2 (13.3%)**	**-**
	**-124/-125 G>A**	**0 (0.0%)**	**0 (0.0%)**	**-**
**BRAF/TERTp**	$BRAF^{wt}/TERTp^{wt}$	13	2	0
	**$BRAF^{mut}/TERTp^{wt}$**	**14 (46.7%)**	**8 (53.3%)**	**-**
	**$BRAF^{wt}/TERTp^{mut}$**	**1 (3.3%)**	**2 (13.3%)**	**-**
	**$BRAF^{mut}/TERTp^{mut}$**	**2 (6.7%)**	**3 (20.0%)**	**1 (n.a.)**
**RAS**	wt	31	15	2[#]
	**RAS^{mut}**	**0 (0.0%)**	**0 (0.0%)**	**(n.a.)**

[#]one distant metastasis only amplified for NRAS; wt: wild-type; mut: mutated; n.a.: Not applicable.
